# Particulate matter characterization of the combustion emissions from agricultural waste products

**DOI:** 10.1016/j.heliyon.2022.e10392

**Published:** 2022-08-22

**Authors:** Sunthorn Laaongnaun, Suthum Patumsawad

**Affiliations:** Department of Mechanical & Aero-space Engineering, Faculty of Engineering, King Mongkut University of Technology North Bangkok, Bangkok 10800, Thailand

**Keywords:** Particles, Emission factors, Fixed bed, Rice husk, Bagasse

## Abstract

This research study examines the levels of particulate matter (PM) which result when two different unprocessed agricultural waste products were burned in a fixed bed combustor under varied air supply conditions. The properties of the PM emissions obtained are: 1) the overall number/mass concentration along with the emission factors (EFs), 2) the distribution of the number and mass size, and 3) the establishment of the morphology of the particles and the trace elements they contain. The findings indicated a total concentration of 3.1 ± 1.9×10^4^ and 9.6 ± 4.5×10^4^ particles/cm^3^ (or number EFs = 3.52×10^9^–2.26×10^10^ and 1.88–5.65×10^10^ particles/kg_fuel_), whereas the total particle mass was found to be in the range of 5.51–10.4 and 1.45–6.08 mg/m^3^ (or mass EFs = 1.58–3.26 g_PM10_/kg_fuel_ and 0.53–3.37 g_PM10_/kg_fuel_) in the case of rice husk and bagasse combustion, respectively. The distribution of the particle sizes was shown to be bimodal for rice husk combustion, but had only a single mode for bagasse combustion. For both fuel types, the predominant particle size emerging during combustion was 0.07μm. From the gravimetric perspective, the dominant sizes were 3.07, 5.13, and 8.09 μm. PM emissions are also affected by the properties of the fuel involved in the combustion process, with ash content, homogeneity of the fuel, and the mineral content all known to be influential factors. It is also understood that the air staging can affect PM emissions during the combustion of rice husks, so a rise in the ratio of secondary air to total air might reduce nanoparticle formation (PM_0.1_) through a shift to the accumulation mode (PM_0.1-1.0_). The results of assessment of the particle shapes and the evaluation of trace elements showed that the particles formed by the combustion of rice husks were predominantly spherical, which can be explained by the dominance of carbon. In contrast, irregular shapes of particle were obtained in coarse-sized particles and the most dominant element is calcium (Ca) and silicon (Si).

## 1 Introduction

Pollution in the atmosphere is predominantly composed of airborne PM (particulate matter), which causes significant harm to human well-being and to the wider environment. It is emitted from various sources, particularly those related to combustion processes for energy production, or transportation use. Moreover, the gradual depletion of the major solid fossil (coal), has aroused the need to research and develop sustainable energy resources. This has revived interest in the use of agricultural residues as alternative fuels. Thailand has an economy which is largely based on agriculture, and accordingly it has significant quantities of agricultural waste products, e.g., empty fruit bunches (EFB) [1, 2, 3], rice straw, bagasse, rice husks etc., which can serve as supplement fuels [4]. However, it is necessary to verify whether the use of such fuels in the combustion process to generate energy will actually result in lower levels of pollution in the form of particulate matter. When coal is burned along with biomass or waste, the resulting PM levels have been investigated. However, the processing usually involves densification followed by combustion in the form of pellets or small bricks [5, 6, 7]. In Thailand, however, the process of lignite co-firing in a fluidized bed along with municipal solid waste (MSW) has been the subject of previous research [8]. In contrast, this study emphasizes gas emissions and the need to achieve efficiency through the optimal combustion conditions, while the generation of PM is of only secondary concern. If focusing on emission concentration of PM from combustion of biomass/coal from literatures, it is noticed that PM emission are varied, according to fuel types, ash content, combustion technology and operating conditions that could be documented briefly as followed. PM emission from firing of wood spruce (0.8% ash content) in fixed bed was 26.5 mg/m^3^ [9], meanwhile combustion of wood pellet (7.6% ash content) emitted 88.85 mg/m^3^ of PM [10], and burning of wood chip in moving grate fired released PM in the range of 60–230 mg/m^3^ [11]. PM emission of 24.5–26.1 mg/m^3^ was found during co-firing of bituminous coal/bagasse in traveling grate boiler [12], while 20–100 mg/m^3^ of PM was observed during co-firing coal/rice straw in pulverized coal fired boiler [13]. Emission factors of particles, in number per fuel weight, when wheat straw, corn straw, or rice straw was burned in stoves was 1.8×10^13^, 1×10^13^, 1.7×10^13^ particle/kg_fuel_, respectively [14] and the rates during combustion in fixed bed combustor, with a high secondary airflow rate (11.4 m^3^/kg_fuel_) of sawdust briquettes, dust-coal briquettes, Coal Wujak and lump wood, were 0.6, 1.4, 9.2 and 2.1 g/kg_fuel_, respectively [15].

In Thailand, co-firing of coal with agricultural residues is deemed as the promising options in both views of economic as decreasing of import coal, and of the abundant of reserve fuels for energy production. However, the study of the process of Thai lignite co-firing with various agricultural residues appears in another section of our work. Highlighting to agricultural residues, finding the best (ready – to use) of unprocessed biomass (e.g., unpelletized or non-briquette, no moisture reduction) is our first attention. For biomass combustion, PM is the most concerned pollutants instead of acidic gases (e.g., sulfur dioxide, nitrogen oxide) because biofuels contain a negligible content of sulfur and nitrogen. To study the relationship between fuel potential and PM emission, we started the pre – burning testing with three residues; 1) EFB (empty fruit bunch), 2) rice husk and 3) bagasse. In early tests, rice husk and bagasse were burned as received, while EFB was cut and shredded to fiber without being neither palletized nor undergone dewatering processes. The preliminary results revealed that the high moisture content (37.7%) of EFB, led to poor combustion, and low bed temperature obtained; 450 °C, and hence incomplete combustion. On the other hand, burning of rice husk and bagasse are easier in operation and produce higher bed temperature, i.e., 750–900 °C with low emission of PM, compared to EFB.

Accordingly, rice husk and bagasse are suitable to further investigation, especially in terms of PM emissions in depth. The current study has accordingly been carried out in order to establish a database covering the emissions of particulate matter from both the qualitative perspective (e.g., mass and number size distribution, particle morphology and its associated trace elements), and quantitative perspective (i.e., PM mass concentration and emission factors) from the combustion of rice husks and bagasse.

## 2 Experimental set-up

### 2.1 Feed materials

Two feed materials used in this study comprise: 1) rice husk and 2) bagasse. These two fuel types were obtained from Nakhon Pathom province in central Thailand, with rice husk coming from a rice mill and bagasse coming from a sugar cane factory. To compare physical properties, the bulk density of bagasse (60 kg/m^3^) is lower than that of rice husk, and takes the form of long-short fibers which have a thick-thin shape, along with a powder component, making difficulty in blending with other feed material, whereas rice husks are very homogeneous in size and shape.

In all testing runs, feed materials were burnt at the same bed height, 84 cm, which mean fuels used per batch had difference in weights and normalized to 1 kg of fuel burned. Feed bulk density of rice husk and bagasse are 90 and 60 kg/m^3^, respectively, which are 0.87 and 0.6 kg_fuel used_/batch. Table 1 illustrates the properties of the two different feed materials.

**Table 1:** The proximate and ultimate analyses of the fuels used and the fuel ash minerals.

	Moisture (%wt.)	Volatile (%wt.)	Fixed Carbon (%wt.)	Ash (%wt.)	Dry-ash-free basis					HHV (MJ/kg)
					C (%wt.)	H (%wt.)	O (%wt.)	N (%wt.)	S (%wt.)	
Rice husk	7.9	59.3	16	16.8	47.7	6.6	46.1	0.4	0.015	14.44
Bagasse	9.9	74.6	15.7	2.3	45.7	6	47.9	0.3	0.015	17.8

Oxide composition of fuel ash (%wt.)	fuel	
	Rice husk	Bagasse

SiO_2_	92.7	42.9
Al_2_O_3_	0.14	23.8
Fe_2_O_3_	2.00	16.9
CaO	0.54	2.20
TiO_2_	0.02	2.50
MgO	0.35	2.10
SO_3_	0.37	0.6
P_2_O_5_	0.43	1.30
Na_2_O	0.07	0.60
MnO_2_	0.19	ND
K_2_O	2.50	3.20

### 2.2 Test rig

**Figure 1:**
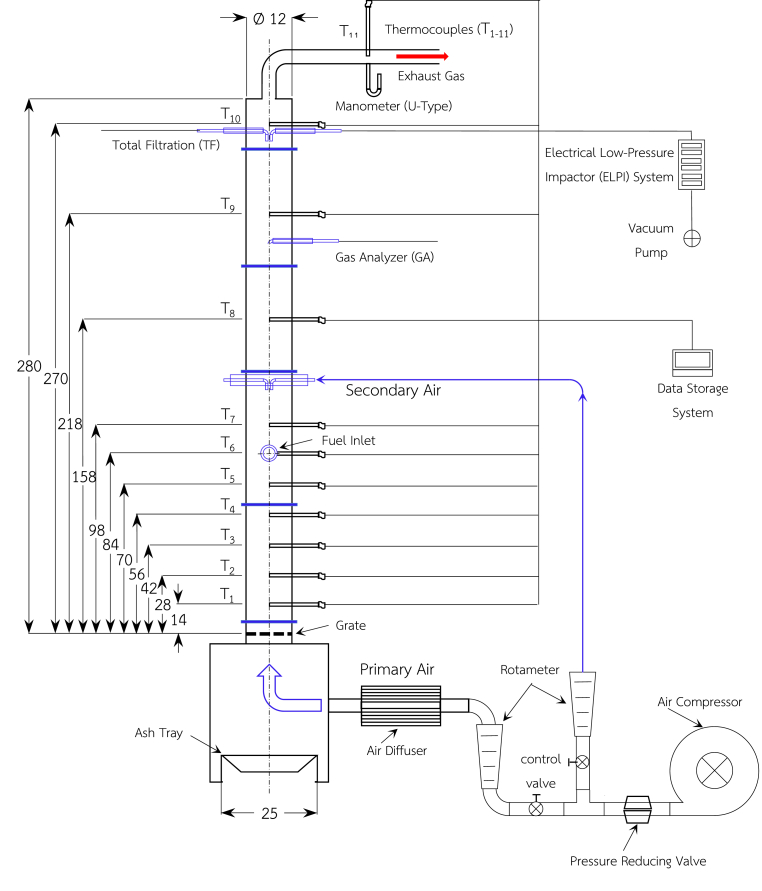
Fixed-bed combustor and measurement systems [16].

A lab-scale fixed bed combustor and associated system were employed to perform the trial runs, as illustrated in Figure 1. The combustor takes the form of a cylinder standing vertically at a height of 280 cm. The size of the internal radius is 6 cm and the insulation layer comprises refractory material (4.5 cm) and rock wool (2 cm). At the base of the combustor is the grate, made from a stainless steels plate containing around 30 small holes of 3 mm diameter. Temperature measurements throughout the reactor were taken using eleven thermocouples (types K chromel-alumel). Six of these thermocouples, designated as T1-T6, set 140 mm apart, measured the bed temperatures, plugged into the combustion zone at a distance of 840 mm from the grate. The remaining five thermocouples, designated as T7-T11 were employed in freeboard to measure the flue gas temperatures. The eleven thermocouples were all linked using a 40-channel Data Storage System (Yokogawa, MV200) in order to show the acquired temperature measurements.

### 2.3 Air supply

The process uses an air supply system which has a single 11-kW two-stage air compressor which also has an air filter. First of all, the air flows into the air regulator, where filters remove any oil or moisture. The air is then separated into the primary and secondary air flows, as can be observed in Figure 1. Two rotameters serve to regulate these two separate air flows. The primary air flow serves as the principal source of combustion air entering the fuel bed. The primary air flow comes into the reactor beneath the fuel bed via the hole in the grate. In contrast, the secondary air flow enters the reactor via the top of the fuel bed at a height of 14 cm. Its role is to react with any gases which leave the fuel bed. In the study of PM emissions, one key consideration to be examined is the effect of the ratio of secondary air to total air. At fixed total flow rate (TA = 0.005 m^3^/sec), the percentage of secondary air was varied from 0 to 30%. By implication, for example when the SA:TA ratio is 30%, this can be considered as combustion under primary airflow: 0.0035 m^3^/sec plus secondary airflow: 0.0015 m^3^/sec.

### 2.4 Particulate sampling and evaluation

The detection of PM emissions on the basis of particle size (0.04 μm–10 μm) was carried out using an Electrical Low-Pressure Impactor (ELPI) with a dilutor system capable of detecting electrical charges and currents on the basis of the particle cut-off diameter. In order to ensure no loss or particles, insertion of the short line of the nozzle probe into the fixed-bed reactor positioned 270 cm from the grate is shown in Figure 1. Two dilutors were used to ensure the sample gas with particles was diluted before it entered the nozzle probe, and subsequently the impactors in the form of 13 stages of non-greasing Teflon filters. Each of the dilutors had a dilution ratio of 1:8, which means 64 times for two dilutors. For sampling, pump operated under a low-pressure condition (vacuum at 100 mbar), corresponding to a sampling rate of 0.01 m^3^/min, to draw PM through filters. For the calculation of parameters concerned: (1) total number concentration, (2) total mass concentration, and 3) emission factors of particle number/mass, the following equations were applied.

(1) $$T_{N}=\overset{s12}{\underset{s1}{\int }}\left(\frac{1}{x}\times \frac{1}{d\;\log\;Dp}\times 64\times c_{m}\right)$$

(2) $$T_{M}=\overset{s12}{\underset{s1}{\int }}\left(\frac{1}{x}\times \frac{1}{d\;\log\;Dp}\times D_{i}^{3}\times \frac{\pi }{6}\times \rho_{p}\times 64\times 0.001\times c_{m}\right)$$

(3) $${\text{ ​ ​ ​EF}}_{\text{n}}=\overset{\text{s}12}{\underset{\text{s}1}{\int }}\left[\frac{1}{\text{x}}\times \frac{1}{d\;\log\;Dp}\times 64\times \left({\text{v}}_{\text{m}}\times 10^{6}\right)\right]/{\text{W}}_{\text{f}}$$

(4) $${\text{ ​ ​ ​EF}}_{\text{m}}=\overset{\text{s}12}{\underset{\text{s}1}{\int }}\left[\frac{1}{\text{x}}\times \frac{1}{d\;\log\;Dp}\times D_{i}^{3}\times \frac{\pi }{6}\times \rho_{p}\times 64\times 0.001\times v_{m}\right]/{\text{W}}_{\text{f}}$$

In Eq. (1), T_N_ is total number concentration (1/cm^3^); C_m_ is a measured current (fA); X is conversion vector (fAcm^3^) that used to calculate particle distribution from the current distribution. Conversion vectors according to each stage were shown in Table 2.

**Table 2:** Conversion vectors using in this study.

Stage	1	2	3	4	5	6	7	8	9	10	11	12
Di	0.0389	0.0705	0.1190	0.2001	0.3139	0.4804	0.7574	1.2238	1.9453	3.0739	5.1343	8.0873
(μm)												
X	0.0091	0.0284	0.0685	0.1403	0.2612	0.4700	0.8811	1.7001	2.8711	4.8166	8.6035	14.3816
(fAcm^3^)												

In Eq. (2), T_M_ is total mass concentration (mg/m^3^); D_i_ is geometric mean diameter of particle (μm); ρ_p_ is particle density (determined at 1 g/cm^3^ under assumption of all particles shaped by spherical). Note: at stage 13^th^ (top impactor), it was used as a pre-cut stage for PM_10_ thus only twelve stages below of impactor (i.e., s1^st^-s12^th^) were used to calculation. In Eq. (3), EF_n_ is particle number emission factors (particle number/kg_fuel_); v_m_ is sample volume (m^3^); W_f_ is weight of fuel used per batch (kg). In Eq. (4), EF_m_ is particle mass emission factors (mg_PM_/kg_fuel_). To validate isokinetic status of particle sampling by ELPI method, Total Filtration (TF) method, was used with ELPI simultaneously. To deal with isokinetic sampling (the velocity of sample gas in a stack is equal to the velocity of sample gas in the sampling probe), particle-laden gas was drawn by pumping air at 0.021 m^3^/min of sampling rate. The sampling probe was made from stainless steel and design to have a 10 mm in diameter of nozzle probe, 30 cm long and 1 mm thickness that resistant to high temperature and corrosion.

In the case of ELPI, using isokinetic sampling similar to the TF method, the initial investigations into stack parameters indicated that the best approximate of nozzle diameter is 8.69 mm (at sampling rate 0.010 m^3^/min), and the following equation was applied.

(5) $$D_{n}=\sqrt{\frac{\left(6.0758\;\times Q_{m}\times P_{m}\right)}{T_{m}\times C_{p}\times \left(1-B_{H_{2}O}\right)}\sqrt{\frac{T_{S}M_{S}}{P_{S}{\left(\Delta P\right)}_{avg}}}}$$

In Eq. (5) where, D_n_ = nozzle diameter (cm); Q_m_ = suction rate through ELPI (10L/min); T_m_= temperature at standard condition (298 ^o^K), P_m_= Pressure at standard condition (760 mmHg), C_p_= coefficient of S type-Pitot tube (0.84), T_s_= the average of absolute temperature of exhaust gas in stack (^o^K), M_s_= dry molecular weight of exhaust gas (29 g/g_mole_), P_s_= the absolute static pressure of exhaust gas in stack (mmHg), B_H2O_= water vapor volume fraction of gas, ΔP_avg_ = Velocity head of exhaust gases across the stack (mmH_2_O).

The analysis of particle geometries and a trace element on the filter, covers the significant modes of the particulate, especially the submicron particle; the nucleation mode (D_i_ = 0.07–0.1 μm or the 2^nd^ stage of the filter) representing the formation of new (fresh) aerosols. The accumulation mode (D_i_ = 0.2–0.31 μm or the fourth stage) indicates the agglomeration and growth of the nanoparticles. The supermicron particle (D_i_ = 3–5 μm or represented by the 10^th^ stage of impactor) representing the unique features of some releasing elements and represents the effective of fuels and of the whole burning processes. Note that the reason to choose D_i_ 3μm instead of the bigger one (D_i_ 5, 8μm) to be the representative is that it deemed as the original size/shape in coarse particle mode that could be linked to the transitions of the larger sized particles. To study particle morphology and its associated trace elements, Scanning Electron Microscope model JSM-6301 assembly was employed in combination with an Energy Dispersive X-ray Spectroscopy (SEM-EDS) model Inca 350 Penta FETX 3. In operation, sample filter was cut approximately 3 × 3 cm^2^ by piece and placing on stub. Carbon and gold were used to induce the conductivity of sample for analysis base on electrical principle. Carbon coating was used for trace elements (e.g., Al, Fe, Si, Ca, etc.) containing in particle, while gold coating was used for total carbon analysis. The spot or area represented the real size of particle was selected on screen followed by firing of electron on that spot, which the transition of energy spectrum (as X-ray) of each element was detected.

## 3 Results and discussion

### 3.1 Effects of fuel types and fuel properties

On the basis of a combustion process using a total air (TA) flow rate of 0.005 m^3^/sec, the total number concentration of particles was 3.1 ± 1.9×10^4^ and 9.6 ± 4.5×10^4^ particles/cm^3^, and the total mass concentration for the particles was found to be in the range of 5.51–10.4 and 1.45–6.08 mg/m^3^ for rice husk combustion and bagasse combustion, respectively. Regarding particle mass concentration, the level of PM emissions produced by rice husks was observed to be greater than was the case for bagasse, as anticipated. This result is according to ash content contained in each residue; 2.3% and 16.8% for bagasse and rice husk, respectively. Therefore, it could be mentioned that levels of PM emission are marked by fuel ash content, the high ash content leads to the greater elutriation of particle-derived ash [17].

In the views of emission factors (EFs), particle number EFs and particle mass EFs in the current research can be observed in Table 3, along with details of the findings from similar investigations in the literature, along with notes on the features of interest from such studies as follows: 1) low bulk density of rice husk could be compensated by their homogeneity to produce low emissions of PM., compared to those particles number/mass generated in some research [18,19,20], 2) EFs of particle number is relatively high in cases of bagasse. Inhomogeneous and low density of them could made void distribution giving a negative impact on the energy density [21], leading to a low combustion temperature obtained, i.e., ≈700 °C and all these mentioned reflecting that at 0.005 m^3^/sec of TA has too high excess air for bagasse combustion. Turn out, too high TA could change the oxygen-limited process to a heat-transfer limited process [22], which means surface reactions between the oxidizer and the carbonaceous surface in the bed is limited, while gas-phase reaction above the top bed may also be disturbed by short residence time, or possibly from air convective cooling, that is not optimized on secondary combustion of volatiles released from the top-bed. This results in the high quantity of vapors/vaporized gases left, which when they condensed and became particles having very small size, always dominated in the overall number.

**Table 3:** Particle number/mass emission factors from combustion of different fuels.

Work/(duplication)	Fuel	Particle number emission factors (particles/kg_fuel_)	Particle Mass emission factors (g_PM_/kg_fuel_)
This study (6 runs)	Rice husk	3.52×10^9^–2.26×10^10^	1.58–3.26
This study (4 runs)	Bagasse	1.88–5.65 × 10^10^	0.53–3.37
Literatures [14]	Rice husk briquette	-	5
Literatures [15]	Birch wood	3.9 ± 0.7 × 10^15^	2.1 ± 0.3
Literatures [16]	Four types of straw	-	4.53–8.75

### 3.2 Particle size distribution

**Figure 2:**
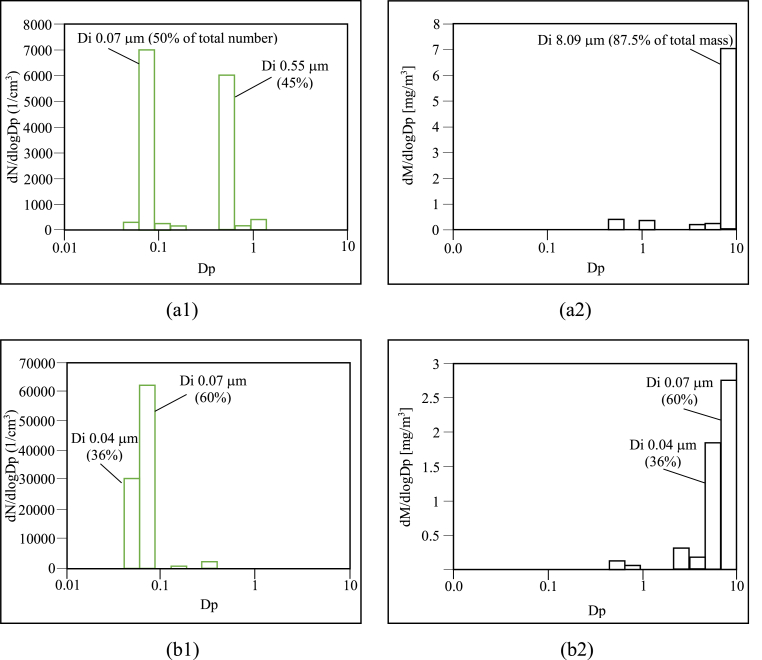
Particle emission distribution in terms of number and mass size following the combustion of (a1, a2) rice husk and (b1, b2) bagasse.

In this stage, the particle emissions could be classified further as PSD (particle size distribution). The findings suggest a bimodal distribution in the case of rice husk, and a single mode for bagasse, as can be observed respectively in Figure 2(a1,a2) and 2 (b1,b2). Figure 2 (a1) is shows total number concentration of principal particle size which arose during the combustion process of rice husk and particle size diameter of 0.07 μm (ultrafine sized-particles) or about 50% of total number. Based on gravimetric or mass consideration, it could be said that the most size range of particles distributing on overall mass concentration of particle were mainly composed of two sizes: 5.13 μm–8.09 μm or coarse particle fraction (PM_1-10_). And Figure 2(b1) is shows total number concentration of principal particle which arose during the combustion process of bagasse and particle size diameter of 0.07 μm (ultrafine sized-particles) or about 60% of total number. Based on gravimetric or mass consideration, it could be said that the most size range of particles distributing on overall mass concentration of particle were mainly composed of two sizes: 3.07 μm–5.13 μm. Higher number concentrations of bagasse generated aerosol in the accumulation mode was due to primary emission of particle in this size ranges, and also because of incomplete combustion in fuel solid biomass. For Figure 2(a2,b2) In contrast, submicrometer-sized particles, especially particles having a diameter size smaller than 0.1 μm, were very much less distributed on overall mass loading although such a given mass was originated by a lot of particle numbers, which indicates that particle size has more influence than particle number in increasing overall mass concentrations.

### 3.3 Influence of air supply

Air partitioning leads to a change in fuel bed conditions into two aspects: 1) the difference of the oxidation state in freeboard and, 2) the difference of the burning rate in bed, in which both factors finally affected particulate emission. The varying effects of changes in air staging were examined in the case of rice husk combustion. Experiments were conducted varying the secondary air to total air percentage, expressed as the SA/TA ratio, using values of 10%, 20%, and 30%, whilst the total air supply (TA) was fixed at 0.005 m^3^/sec.

**Figure 3:**
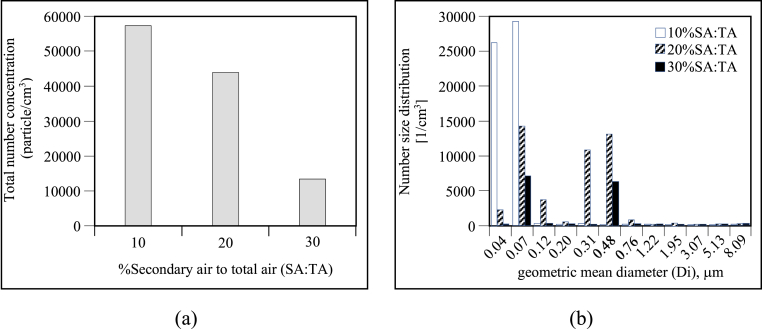
Particle emissions after rice husk combustion using varied SA/TA ratios: a) total number concentration, and b) number size distribution.

Figure 3(a) showed that total number concentrations were, respectively, 5.6 × 10^4^, 4.6 × 10^4^ and 1.4 × 10^4^ particle/cm^3^ for the SA/TA ratios of 10%, 20% and 30% respectively. In addition, Figure 3(b) reveals that at the 10% ratio, the smallest particle size was predominantly formed (D_i_ 0.04–0.07μm; ∼98% of the total), which appeared to be the consequence of an incomplete process of oxidization during the gas phase, suggesting that if the secondary air flow above the fuel bed is increased, the result will be more complete oxidization of the gases which leave the fuel bed, which the resulting reduction in the PM emission formation. Furthermore, Figure 3(b) also indicates that if the secondary air flow is raised to 20% or 30%, the average particle size will be altered so that the accumulation mode (PM_0.1-1_) will prevail over the ultrafine mode (PM_0.1_) in terms of particle size, especially in the case of particle sizes in the range of 0.31–0.48μm. It was shown that 62% and 47% of the total particle number were respectively contained in the accumulation mode at 20% and 30% for secondary air*.* It has also been reported that increased PM sizes might result from coagulation and lower temperatures [23]. Our findings would support such a view, as the average temperatures of the bed were 800 °C, 750 °C, and 700 °C, when the secondary air ratio percentages were respectively 10%, 20%, and 30% of secondary air. Therefore, it can be argued that a high SA/TA ratio (20% and 30%) could lead to the increased availability of seed on which to base the coagulation of particles, which became apparent from the larger average diameter of the particles combined with the slight suppression in the formation of the smallest ultrafine particles (i.e., D_i_ 0.04–0.07μm).

### 3.4 Morphology of the particles

**Figure 4:**
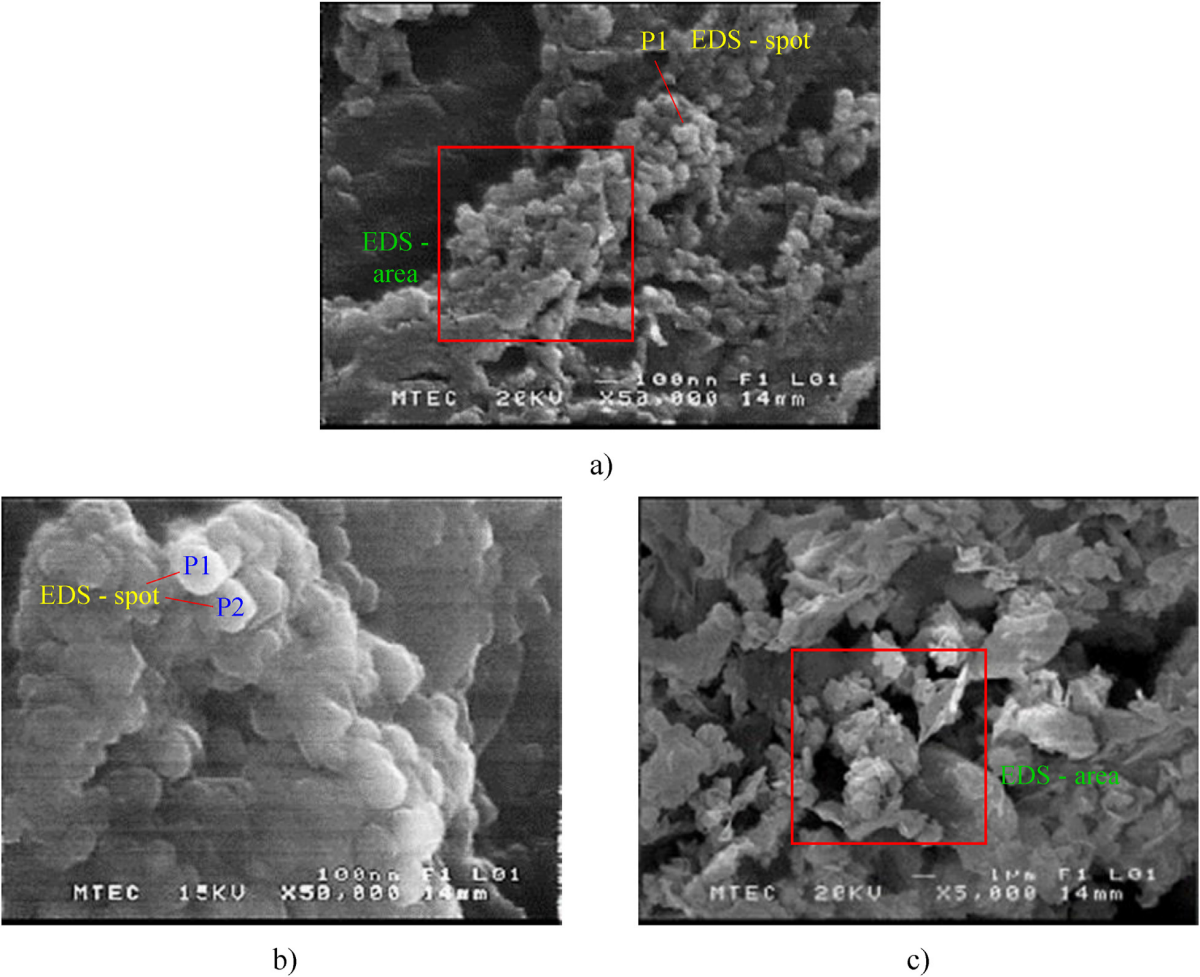
Microstructures of particles derived from rice husk combustion: a) rice husk-0.07 μm, 50,000X, b) rice husk-0.2μm, 50,000X and c) rice husk-3.0 μm, 5,000.

The particle geometry was examined via SEM. Figures 4(a) and 4(b) indicate that submicrometer-sized particles, e.g., D_i_ 0.07, 0.2 μm, produced during rice husk combustion are mostly characterized by spherical shape either as isolate one or as grape – like and/or chain – agglomerate. This confirms that the most particles are mainly formed through a condensation, followed by coagulation process, i.e., gas – to – particle pathways. This is certainly caused by the enrichment of volatiles in the rice husks and, thus devolatilization is the most important of all combustion processes.

On contrary, it can be noticed in Figure 4 c) that coarse fly ashes (e.g., D_i_ 3μm) are not typified merely by a round, rod, or cluster shape but also by the layered or filmed structures, containing microspheres in various sizes and. It should be noted that the layer structure, as coarse fly ash, looks like to the fresh rice husks before burning, which mean that there has been some loss of fresh fuel during its burning, caused by the entrainment directly of mineral from the bed.

### 3.5 Chemical composition

The chemical compositions obtained from EDS analysis are shown in Table 4, according to the selected spots or areas or both of them in Figure 4(a-c) for specific certain sizes (i.e., 0.07μm, 0.2 μm, 3.0 μm).

**Table 4:** Chemical compositions in the various sizes of particle during rice husk combustion.

	Rice husk		
Geometric Mean Diameter (GMD)	0.07μm	0.2μm	3.0μm
Chemical (% wt.)			
Total carbon	86.5	90.2	21.5
Na	0.1		
Mg	0.4	0.2	1.0
Al	3.0	2.8	
Si	4.1	4.6	17.8
S			0.7
Cl		0.1	5.8
K	0.4	0.3	
Fe	5.2	1.1	4.1
Ca		0.7	49.2
Ti	0.1		

Note: only the significant elements will be underlined, in order to extend their role in particle structure, and the enrichment in each size of particle.

In case of rice husk combustion, the main observation from Table 4 is that ultrafine and fine particles (e.g., D_i_ 0.07, 0.2μm) are considerably enriched by total carbon together with a minor content of iron (Fe) and aluminium (Al) according to Figure 4a) and b). This could be said that the unburned carbon (soot) was typified by spherical shape, suggesting that they nucleated after burning or out of the hot zone. For coarse particles e.g., D_i_ 3.0 μm, it is clearly noticed that calcium (Ca) is the most enriching element (49.2% wt.) and followed by silicon (17.8% wt.). In fact, rice husk contains only 0.54%wt. of calcium oxide (CaO), but 92.7%wt of silica (SiO_2_) as oxide compositions of fuel ash (see. Table 1.), Ca seems accordingly to be the commonest element in coarse particles. Because Ca is the specific mineral inclusion, that does not volatilize during combustion [24] so that it mostly disintegrated directly from the fuel bed due to thermal – defragmentation. Regarding to the relationship between coarse particle shapes and it associated trace element, it can be seen that the general structure of Ca is governed by irregular shapes as shown in Figure 4c). Moreover, the unique properties of Ca, apart from the originally large size, is that Ca has vicinity or essential to bonding fly ash each other, so does the Silicon (Si). Unless silicon will cover in various sizes of particle, which means Si could either be volatilized during burning and condensed to fresh particles containing – Si, e.g., D_i_ 0.07 μm, or Si could act as the core – media for the particle growth e.g., D_i_ 0.2, 3.0 μm. As mentioned earlier, it is clearly apparent that Ca and Si are important factors when considering the greater particle size in terms of average diameter.

## 4 Conclusion

This study examined the combustion of unprocessed agricultural waste products, namely rice husk and bagasse, in experiments which used a fixed bed combustor to produce a database detailing the resulting emissions of particulate matter. The main conclusions can be documented as follow:

1) The non-briquette rice husk could be burned effectively at 0.005 m^3^/sec of total airflow (TA) rate with low emissions of PM. The homogeneity of rice husk is more pronounce over the light weight of them to complete combustion. For bagasse, the optimum (lower) of TA is needed, and densification processes is necessary.
2) The level of PM emission is drastically marked by fuel ash content; the high ash leads to the most elutriation of particle-derived ash. In addition, if the fuel (i.e., bagasse) contains inhomogeneity and bulk density is low (i.e., 60 kg/m^3^), it will generate a negative effect on energy density, leading to a low combustion temperature obtained, finally leads to high emissions of PM, especially in terms of number. However, in the case of rice husks, though it contains low bulk density (90 kg/m^3^) but being homogeneous itself (in size and shape), particulate emissions will be low.
3) Particle size distribution (PSD) in terms of number, PSD from rice husk combustion is characterized by bimodal distribution (D_i_ 0.07 and 0.55μm), suggesting that rice husk burning has a high tendency to produce the accumulation particle (0.1μm < Di < 1 μm), which tends to reduce the high formation of nucleated sized-particle (0.04μm < Di < 0.1 μm). In addition, PSD from bagasse is marked by single-modal. In the rice husk and bagasse combustion processes, the principal particle size which emerged in both cases was the same, at 0.07 μm. Base on gravimetric or mass consideration, it could be said that the most size range of particles distributing on overall mass concentration of particle are mainly composed of three sizes: 3.07, 5.13, and 8.09 μm, though such a given mass is originated by a small particle number suggesting that particle size has more influence than particle number in increasing overall mass concentrations.
4) The effects of the differences in air supply on particulate emissions can be concluded as follows: when the secondary air flow over the fuel bed was increased to a greater SA/TA ratio, the evaporated-volatiles/reduced gases which were released from the fuel bed would be more fully oxidized, thus lowering the levels of PM emissions, and in particular reducing the level of ultrafine-sized particles. Furthermore, sufficient excess air in the bed and sufficient total excess air will enhance burning rate and complete combustion, resulting to low emissions of particulate matter.
5) For particle morphology and its associated element, it is observed that particle shapes from combustion of rice husk is mainly characterized by spherical shapes. In addition, fine particles that marked by spherical shape will always correspond to the high content of total carbon. On the other hand, coarse particles that typified by irregular shape, will always relate to the high content of Calcium and Silicon.

## Declarations

### Author contribution statement

Sunthorn LAAONGNAUN: Conceived and designed the experiments; Performed the experiments; Analyzed and interpreted the data; Contributed reagents, materials, analysis tools or data; Wrote the paper.

Suthum PATUMSAWAD: Conceived and designed the experiments; Analyzed and interpreted the data; Wrote the paper.

### Funding statement

This work was supported by the Department of Mechanical & Aerospace Engineering at 10.13039/501100007345King Mongkut's University of Technology North Bangkok.

### Data availability statement

Data will be made available on request.

### Declaration of interests statement

The authors declare no conflict of interest.

### Additional information

No additional information is available for this paper.
